# Chest Pain: A Relatively Benign Symptom of Type IV Hiatal Hernia

**DOI:** 10.7759/cureus.11459

**Published:** 2020-11-12

**Authors:** Muhammad Z Khan, Hamza Yousaf, Dushyant S Dahiya, Farah Wani, Asim Kichloo

**Affiliations:** 1 Internal Medicine, Central Michigan University College of Medicine, Saginaw, USA; 2 Internal Medicine, Nishtar Medical University, Multan, PAK; 3 Family Medicine, Samaritan Medical Center, Watertown, USA

**Keywords:** hiatal hernia, gastroesophageal reflux disease, chest pain, omeprazole, thorax

## Abstract

Hiatal hernia results from the translocation of intra-abdominal contents from their usual position into the thorax. They can be categorized into type I-IV which implies varying gradations of herniation. The symptomatology can range from just chest pain in the less severe types to respiratory and hemodynamic compromise resulting from strangulation in the advanced hernias. Our patient was an 81-year-old female with a past medical history of gastroesophageal reflux disease (GERD), deep venous thrombosis (DVT), hypertension, hyperlipidemia, coronary artery disease (CAD), and cerebrovascular accident (CVA), who presented to the emergency department (ED) with the chief complaint of chest pain. Assessment of the vitals in the ED revealed a temperature of 37.2 °C, respiratory rate of 18 breaths/minute with an oxygen saturation of 100% on room air, heart rate of 95 beats/min, and blood pressure reading of 132/110 mmHg. Due to significant concern of a possible coronary pathology leading to chest pain, the patient was given 325 mg of aspirin and one tablet of sublingual nitroglycerin. Her electrocardiogram (EKG) was unremarkable but the chest X-ray revealed a large retrocardiac hernia. The finding was corroborated after a review of the computerized tomography (CT) scan performed at the outlying facility. She was treated with omeprazole, a gastroenterologist was consulted, and an esophagogastroduodenoscopy (EGD) performed which revealed significant erosions in the distal esophagus and gastric antrum. She was deemed a high-risk surgical candidate for any intervention and thus managed conservatively with proton pump inhibitor (PPI) therapy. The case highlights the pertinent facts about hiatal hernia. Although the diagnosis of chest pain with the aforementioned comorbidities could be skewed towards coronary pathology, keeping a wide differential is important so that the right diagnosis can be made in a timely fashion and complications avoided.

## Introduction

Hiatal hernia is characterized by the displacement of abdominal contents, mainly the stomach and abdominal viscera into the thoracic cavity through the diaphragmatic opening. It can be further classified into four distinct subtypes depending on the grade of displacement of the abdominal contents. The clinical presentation and the severity of symptoms vary significantly depending on the subtype of hiatal hernia. Type I hernia involves the displacement of the gastroesophageal junction into the thoracic cavity and is also known as sliding hiatal hernia (SHH), whereas types II-IV involve translocation of the gastric fundus and other intra-abdominal contents. These are also known as para-esophageal or rolling hernias. In patients with type I hiatal hernia, the signs and symptoms are mainly due to the reflux of the gastric contents into the esophagus. However, more severe complications such as strangulation of the herniated abdominal content and perforations secondary to gastric ulcers are usually seen with the more advanced subtypes (II-IV) [1,2]. The mainstay treatment for hiatal hernia is elective surgical repair as it has better patient-centered outcomes, but recurrences are known to occur [3]. Medical management via the use of proton pump inhibitors (PPIs) plays an essential role to limit mucosal injury and promote healing. In this case report, we primarily focus on the diagnostic approach and management of hiatal hernias.

## Case presentation

**Figure 1:**
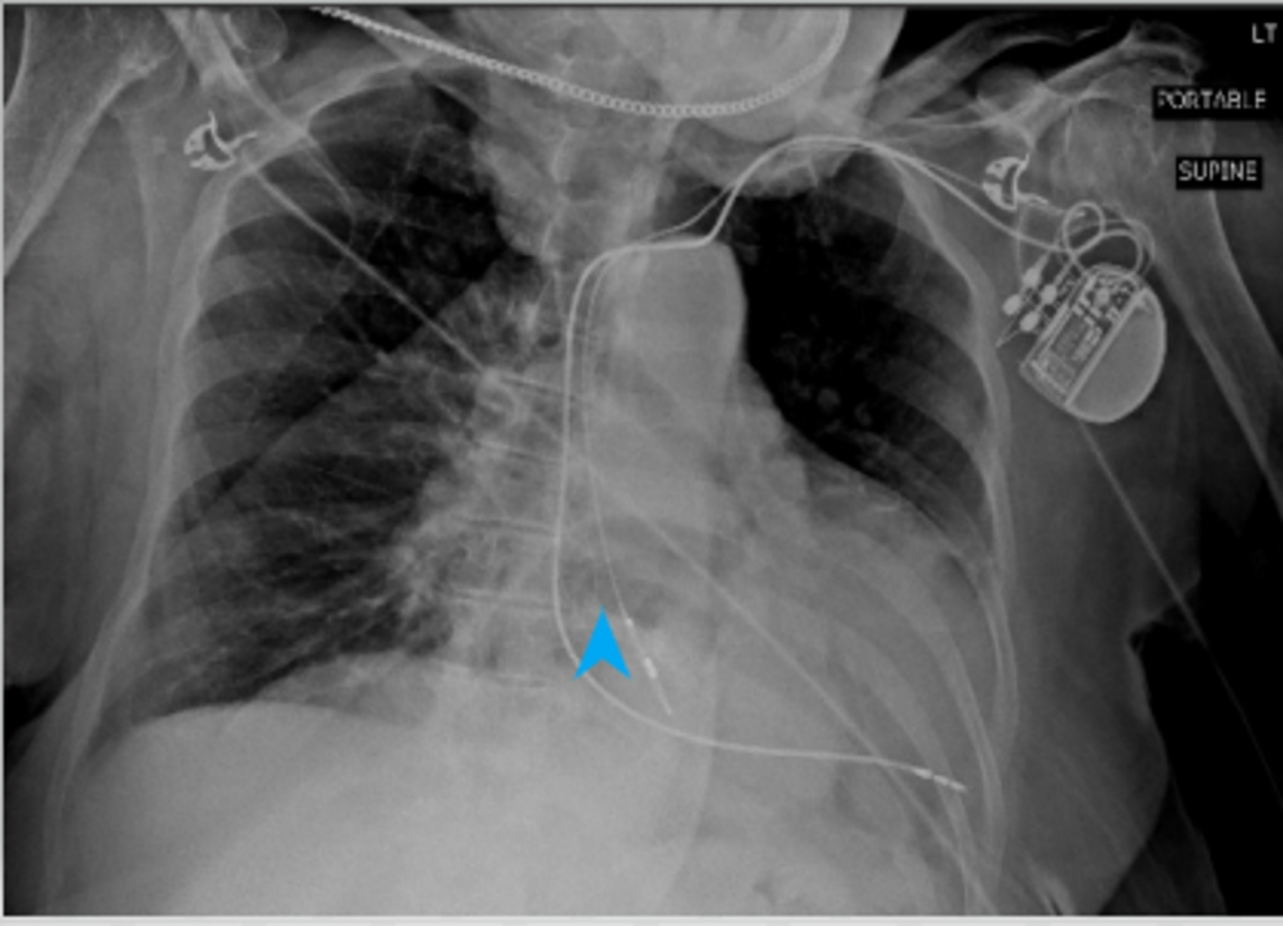
Anteroposterior view chest X-ray. The arrowhead pointing at the gastric shadow in the thoracic cavity.

**Figure 2:**
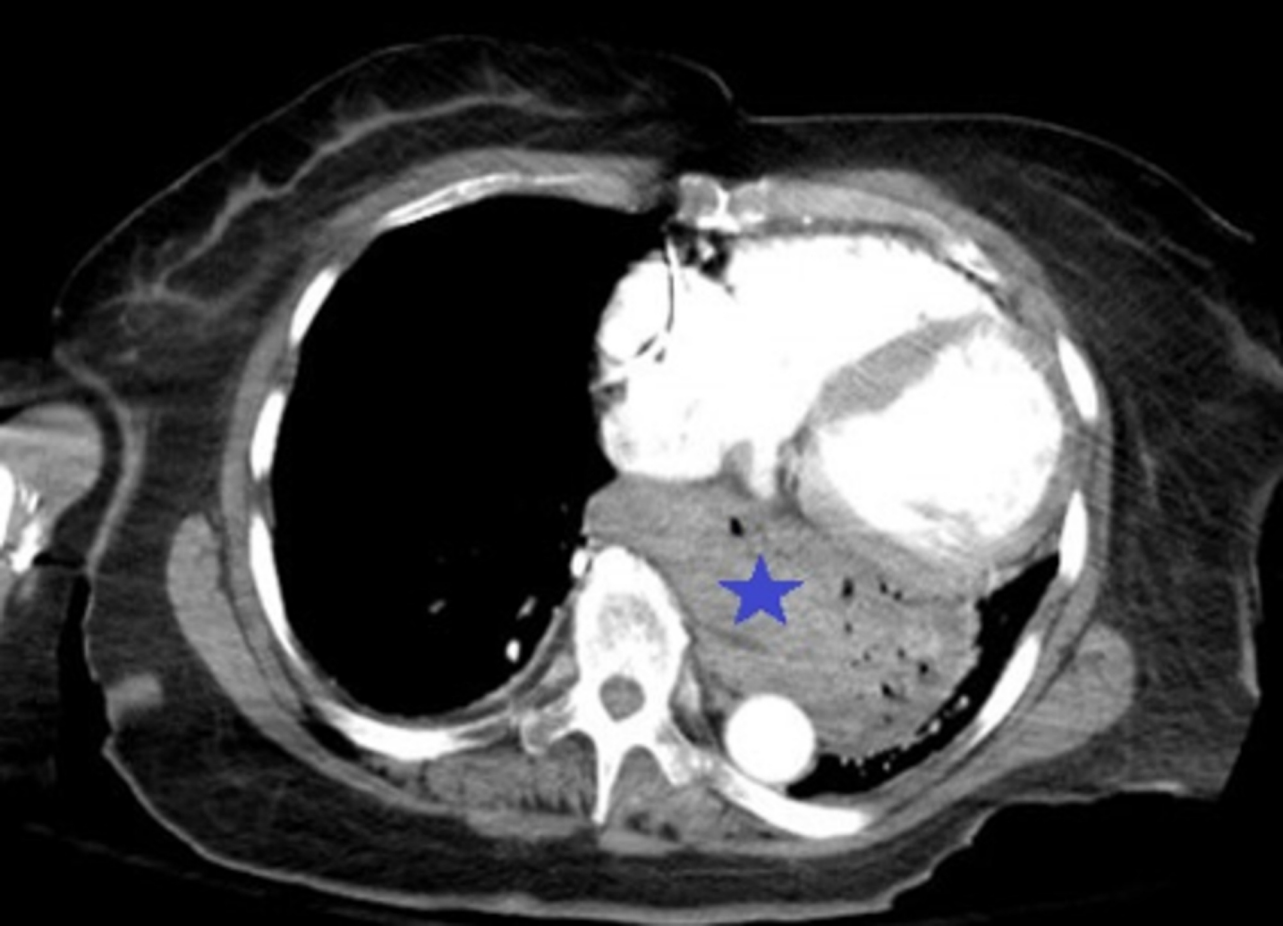
CT image reveals the findings An axial view CT scan of the chest showing the presence of a hiatal hernia (as indicated by the blue star).

We present the case of an 81-year-old woman with a past medical history of gastroesophageal reflux disease (GERD), deep venous thrombosis (DVT), hypertension, hyperlipidemia, coronary artery disease (CAD), and cerebrovascular accident (CVA), who presented to the emergency department (ED) with chest pain. The patient characterized the pain as sharp, of moderate intensity, and localized mainly to the retrosternal region without radiation. She reports worsening of the pain on respiration and movement without any specific relieving factors. Assessment of the vitals in the ED revealed a temperature of 37.2 °C, respiratory rate of 18 breaths/minute with an oxygen saturation of 100% on room air, heart rate of 95 beats/min, and blood pressure of 132/110 mmHg. Due to significant concern of a possible coronary pathology leading to chest pain, the patient was given 325 mg of Aspirin and one tablet of sublingual nitroglycerine. A Stat electrocardiogram (EKG) and chest X-ray were performed. The EKG revealed a left-axis deviation without ST-T segment changes or abnormalities. Troponin levels were tested and found to be within normal limits. Other laboratory investigations such as complete blood count (CBC) and comprehensive metabolic panel (CMP) were unremarkable. Upon further discussion with the patient, she reported having intermittent retrosternal burning sensation with a metallic taste in her mouth. The chest X-ray revealed the presence of a possible large retrocardiac hernia (Figure 1). The patient reported having a CT scan at an outlying facility and after reviewing the computerized tomography (CT) images (Figure 2), a diagnosis of a hiatal hernia in the retrocardiac area was established. The patient was admitted to the hospital and started on pantoprazole 40 mg twice daily. Gastroenterology was consulted, and an esophagogastroduodenoscopy (EGD) was planned. The findings on EGD were consistent with a diagnosis of grade IV hiatal hernia with numerous ulcerations in the distal esophagus and antral gastritis. Biopsy of the area revealed chronic atrophic gastritis, and the biopsy specimens were negative for *Helicobacter pylori* infection. Eventually, surgery was consulted for possible intervention, but the patient was deemed a high-risk surgical candidate due to the presence of multiple comorbidities. Hence, a decision was made for conservative management. The patient was continued on pantoprazole and showed significant improvement in her symptoms over the next day. She was eventually discharged on pantoprazole 40 mg twice daily along with metoclopramide to improve gastric motility. She was encouraged to have a repeat EGD for surveillance in one year.

## Discussion

Para-esophageal hernia (PEH), SHH, giant hiatal hernia (GHH), large hiatal hernia (LHH), intrathoracic stomach (ITS), and upside-down stomach (UDS) are common terminologies often used interchangeably to describe hiatal hernia and signify the disease spectrum [3]. Hiatal hernias can be classified into four distinct subtypes (types I-IV) based on the structures displaced through the esophageal opening of the diaphragm into the mediastinum. Type I hernias, also known as SHH, account for approximately 90% of the cases while the incidence of types II-IV varies between 5% and 10% [4] in patients. Type I hiatal hernias tend to be less severe in intensity with minimal complications; however, the complication rate is high for the latter types. The proposed mechanism for the development of hiatal hernia includes raised intra-abdominal pressure secondary to other causes or widening of the esophageal hiatus due to an acquired or congenital defect in the diaphragmatic opening [5]. GERD is commonly associated with hiatal hernia and the incidence rate increases significantly with an increase in age [6]. The phreno-esophageal membrane acts as a support for the esophageal hiatus; however, with the increasing age, a decrease in elastic component of the tissue results in the laxity of this membrane thereby explaining the increased incidence of hiatal hernias in the elderly [7].

Establishing a diagnosis of hiatal hernia at an early stage helps improve outcomes in the management of the disease. Numerous imaging modalities such as barium swallow, CT, and an EGD can be employed to establish a diagnosis and help classify the type of hiatal hernia. A ≥2 cm distance between the B-line (an anatomical landmark) and the diaphragmatic hiatus is required for the diagnosis of type I hernia [8]. EGD offers an advantage over other investigations as it helps with direct visualization and gradation of the sliding hernia during a retroflexed position. However, sometimes type IV hernias may go undetected on an EGD thereby reflecting a major disadvantage [9]. High-resolution manometry (HRM) with the determination of the pressure gradients can also help identify subtle variations in the reflux phenomenon leading to accurate diagnosis of the sliding hernia [8]. HRM has a higher sensitivity (94.3%) and specificity (91.5%) for the detection of hiatal hernias when compared to barium swallow or endoscopic modalities [10]. CT scan is an adjunct to diagnosis as it may help determine the complications associated with hiatal hernia such as pneumomediastinum, pneumoperitoneum, and gastric volvulus [11]. In a pre-operative setting, all of these diagnostic modalities have roles to play and can be used in combination with each other [12].

In patients with uncomplicated hiatal hernia, the treatment strategy is focused on symptom control. Lifestyle modification such as weight loss, dietary compliance, and appropriate meal timings play a key role. The American College of Gastroenterology (ACG) recommends a twice-daily therapy with PPIs for a period of eight weeks [13]. Prokinetic agents do not have a major role in the management of hiatal hernias [4]. Surgical intervention is reserved for large hernias, PEHs with recurring symptoms, and in patients presenting with clinical features of incarceration or strangulation of the abdominal contents [14]. Additionally, the symptoms of advanced types (II-IV) of hernias warrant immediate evaluation and intervention due to the significant risk of complications [1,12]. For patients with hiatal hernia, the clinical presentation with only chest pain and reflux is not frequently seen. In our case, the patient presented with chest pain, and there were significant concerns for an acute coronary pathology as the underlying cause; however, further investigations revealed the presence of a type IV hiatal hernia. Patients presenting with large hernias but minimal symptoms may be observed over time and invasive surgical intervention is indicated only when the symptoms worsen [12]. Surgery for type III and IV hernias is usually extensive and may be associated with a high rate of complication and risk of recurrence [15]. Furthermore, with the advent of newer laparoscopic techniques, surgical treatment for hiatal hernias is relatively safe with fewer complications [16]. Moreover, in patients who are candidates for bariatric surgery, laparoscopic sleeve gastrectomy can be performed simultaneously with the hernia repair and has additional advantages as proposed in the study conducted by Mahawar et al. [17]. Laparoscopic fundoplication is currently the most frequently used procedure alongside surgical repair to control the symptoms of reflux [4]. Manometry helps to quantify the degree of reflux which should be taken into account before a planned fundoplication [15,16]. In conclusion, surgery offers significant benefits to patients in a younger demographic, those with fewer comorbidities who are able to tolerate the surgical intervention, bariatric patients with a BMI > 35 kg/m^2,^ and elderly patients with refractory symptoms [12].

## Conclusions

Gastroesophageal reflux is the most common symptom associated with all the types of hiatal hernia. Management of reflux is focused on the use of PPIs. Larger hernias can often strangulate and may lead to varying symptoms. Advanced hiatal hernias presenting with only chest pain and reflux as the only presentation are seen less frequently. In patients with higher risk stratification, the diagnosis of chest pain may be skewed towards an acute coronary syndrome. Patients with type I hiatal hernia may be managed with oral PPIs; however, surgical intervention is usually indicated for the advanced types. Additionally, patients who are high-risk surgical candidates are usually managed conservatively to prevent adverse outcomes of the surgical intervention.
